# Occlusion, jaw function and nocturnal muscle tone in obstructive sleep apnea with and without sleep bruxism

**DOI:** 10.1007/s00784-025-06454-7

**Published:** 2025-07-09

**Authors:** Janine Sambale, Ulrich Koehler, Regina Conradt, Karl Kesper, Werner Cassel, Mikail Degerli, Christian Viniol, Heike Maria Korbmacher-Steiner

**Affiliations:** 1 Department of Orthodontics Clinic of Dentistry, Philipps-University Marburg, Georg-Voigt-Str. 3, 35039 Marburg, Germany; 2 Department of Pneumology, Philipps-University Marburg, Marburg, Germany; 3 Faculty of Health Sciences, University of Applied Sciences Gießen, Berlin, Germany

**Keywords:** Obstructive sleep apnea, Sleep bruxism, Electromyography, Muscle tone, Jaw function, Occlusion

## Abstract

**Objectives:**

Sleep bruxism (SB) is highly prevalent among patients with obstructive sleep apnea (OSA), yet its etiology remains unclear. This prospective clinical trial aimed to evaluate the diagnostic relevance of occlusion, jaw function, and electromyographic (EMG) muscle tone in OSA patients with and without SB.

**Materials and methods:**

A total of 105 OSA patients (74 males, 31 females; mean age: 56.1 ± 11.4 years) were assessed, including those with SB and without SB (NSB). Evaluations included occlusal parameters, the Jaw Functional Limitation Scale (JFLS-20), and polysomnography with EMG muscle tone analysis. Descriptive statistics, inter-group comparisons, Spearman’s correlation analyses, and Receiver Operating Characteristic (ROC) curve analyses were performed.

**Results:**

No significant differences in occlusal parameters were observed between the SB and NSB groups. However, SB patients exhibited significantly higher JFLS-20 scores compared to NSB patients (*P* = 0.002; mean global score: 20.79 ± 31.96 vs. 6.52 ± 9.70). EMG muscle tone showed significant correlations with JFLS mobility (*P* = 0.015) and overall jaw function (*P* = 0.046). ROC curve analysis for EMG muscle tone revealed an Area Under the Curve (AUC) of 0.911 (*P* < 0.001). An optimal cutoff value of 9.79 µV for EMG muscle tone demonstrated a sensitivity of 78.6% and specificity of 87.9%.

**Conclusion:**

EMG muscle tone may serve as a preliminary reference point for differentiating SB from NSB in OSA patients, whereas occlusion lacks diagnostic significance.

**Clinical relevance:**

These findings highlight the importance of incorporating functional assessments into the diagnostic workflow for SB in OSA patients. Therapeutic strategies should prioritize functional management rather than occlusal corrections.

## Introduction

The relationship between obstructive sleep apnea (OSA) and sleep bruxism (SB) is a topic of growing scientific interest due to the significant impact of OSA on overall health [1].

OSA is characterized by recurrent episodes of partial or complete upper airway obstruction during sleep, which can lead to cardiovascular and metabolic complications [2, 3]. The high prevalence of OSA underscores the importance of investigating its associations with comorbid conditions such as SB [1, 4].

SB is defined as masticatory muscle activity during sleep, which can be rhythmic (phasic) or non-rhythmic (tonic). In otherwise healthy individuals, SB is not considered a movement or sleep disorder [5]. In healthy subjects, SB presents with a prevalence of 12–13% and is primarily triggered by anxiety, psychological stress, or use of exogenous substances such as alcohol and nicotine [6–9]. Despite the etiology not yet being fully understood, SB appears more common in patients with OSA (26 − 54%), suggesting a possible link that requires further research [5, 7, 10].

SB may have both detrimental and potentially adaptive effects. Its negative impact on oral health includes abnormal tooth wear and temporomandibular disorders (TMD), which can reduce quality of life [11–13].

Interestingly, SB does not always present with typical TMD pain symptoms but is associated with musculoskeletal complaints such as stiffness, tenderness, or reduced jaw mobility [14]. Recent findings suggest that these non-painful symptoms may precede TMD pain, underscoring the need to consider such early signs in future SB research [15]. Despite the recognized importance of orofacial function in overall health, it remains underreported in the context of SB among sleep apnea patients [16].

Peripheral factors such as occlusion or craniofacial morphology are currently considered as secondary factors for SB. Although occlusal parameters have often been studied in self-reported SB, their role in clinically detected SB among OSA patients has not yet been investigated [17]. This underscores a gap in understanding the potential influence of occlusion in this specific cohort, particularly given that SB may have a distinct etiology in OSA patients compared to healthy individuals.

In 1996, Lavigne et al. [18] introduced the Research Diagnostic Criteria for SB (RDC-SB) based on rhythmic masticatory muscle activities (RMMAs), yet findings regarding SB activity remain inconsistent. While some authors reported high variability for SB activity, others found stable SB diagnosis over several nights [19–22]. To address this variability in RMMAs and in bruxism episode index (BEI), the present study focused on analyzing nocturnal electromyographic (EMG) masseter muscle tone.

The physiological state of muscle tone plays a pivotal role in understanding motor dysfunctions, such as SB. Normal muscle tone is characterized by a slight, consistent resistance that facilitates proper motor function. In contrast, hypertonia, an abnormal increase in muscle tone, can result in excessive muscle tension and functional impairment, leading to orofacial pain and TMD. Therefore, the assessment of muscle tone is essential for accurate clinical diagnosis and therapeutic treatment and responses in patients with OSA [23].

Currently, no established EMG thresholds exist for SB in OSA. Some studies reported increased nocturnal muscle tone in OSA patients with SB [24]. Moreover, Raphael et al. [25] found elevated EMG levels during non-RMMA periods in TMD patients, prompting the question of whether a cut-off value might exist for EMG tone in SB. Given the close link between SB and TMD, this requires further study [26, 27].

This study aims to evaluate and improve diagnostic criteria and, indirectly, the treatment of SB in OSA patients. The focus is on assessing jaw function, examining occlusal characteristics, and analyzing nocturnal muscle tone for clinically detected SB.

Therefore, the primary objectives are as follows:

- Evaluating the relevance of jaw function and occlusion in the diagnosis of SB in OSA.
- Establishing a cut-off value for EMG muscle tone that differentiates patients with OSA with and without SB.

We hypothesized that functional parameters, including the nocturnal muscle tone and jaw functional limitations, play a more significant role in the diagnosis of SB among OSA patients rather than the static occlusal parameters.

## Methods

### Study design and subjects

This analysis is part of a larger project and represents a secondary analysis of our previous published prospective clinical trial [24]. The study was approved by the Ethics Committee of Philipps-University Marburg (reg. no. 13/22–2022) and registered at the “German Clinical Trial Register, DRKS” (DRKS0002959). A detailed study protocol was developed prior to the start of the study. The design of the protocol was guided by the SPIRIT guidelines [28, 29]. The principal investigator (JS) is a certified investigator according to Good Clinical Practice (GCP), and all relevant GCP standards, including informed consent procedures and trial registration, were followed.

The dataset included 105 patients with OSA underwent clinical and polysomnographic intervention. Inclusion criteria were OSA with an apnea–hypopnea index (AHI) ≥ 10 events/hour and a minimum of 4 h total sleep time (TST) confirmed by the stationary PSG, age > 18 years, permanent dentition, and number of teeth > 20. The exclusion criteria were lack of patient’s willingness to sign an informed consent form, any neurological, psychiatric or sleep disorders other than obstructive sleep apnea, neuropathic pain with medication intake and impairment of muscle and respiratory function and psychoactive medication intake with risk of jaw muscle and/or limb activity; and patients with removable dentures [24].

The sample size was determined based on the primary objective of the overall project, which was to estimate the prevalence of SB in OSA patients. The sample size was 100 patients, calculated based on an assumed prevalence of 33%, a 95% confidence interval, and a margin of error of ± 0.092 (resulting in a CI width of 0.184).

SB diagnosis was based on the anamnestic and clinical examination according to American Academy of Sleep Medicine (AASM) criteria [2].

Patients with probable SB reported at least one anamnestic symptom: awareness of painful, tiredness, stiffness and limited or painful jaw opening upon awakening, morning headache and/or tooth grinding noises occurring at least 3–5 nights per week over 6 months reported by a sleep partner and at least one clinical sign: tooth wear according to Wetselaar & Lobbezoo [30] (grade > 1) and hypertrophy of the masseter muscle. The criteria used to categorize individuals into either the sleep bruxism (SB) or non-sleep bruxism (NSB) group are detailed in Table 1.

**Table 1:** Protocol for group assignment

Diagnostic	Diagnostic result			
Anamnestic symptoms*	No	Yes	No	Yes
Clinical symptoms*	No	No	Yes	Yes
Group assignment	NSB	NSB**	NSB***	SB

Abbr.: SB = sleep bruxism, NSB = non-sleep bruxism

*At least one anamnestic and one clinical symptom based on AASM criteria [1]

**Patient overestimated anamnestic symptoms

***Suspected wake bruxism

Furthermore, each patient filled in a DC/TMD symptomatic questionnaire and Jaw Functional Limited Scale (JFLS-20) [31, 32].

### Jaw function

Jaw function was diagnosed with the Jaw Functional Limitation Scale (JFLS), which is an instrumental tool for evaluating functional limitations of the orofacial system. The authors used the longer version with 20 items (JFLS-20). It comprises three subscales with numeric scales ranging from 0 (no limitation) to 10 (severe limitation). These subscale scores can be calculated as the sum of functional limitation of mastication (items 1–6), vertical jaw mobility (7–10), and communication described by emotional and verbal expression (13–20). The global score was derived by summing the scores of the three subscale scores [32, 33].

### Clinical examination

The clinical examination was performed by a trained orthodontist (JS). To assess occlusal characteristics, patients were scanned using the 3Shape TRIOS 4^®^ intraoral scanner. The anteroposterior relationship of the first molars was analyzed, and in the absence of first molars, the canines were assessed.

Following Grünberg’s approach, reconstructions of the first molars and canines in the upper and lower jaws were performed in cases where signs or symptoms of mesial drift were observed [34]. This approach represents a dental approximation for skeletal classification, but does not constitute a cephalometrically validated skeletal analysis.

After reconstruction Class I was defined by the mesiobuccal cusp of the maxillary first molar occluding in or near the mesiobuccal groove of the mandibular first molar. Class II.1 was characterized by at least an end-to-end class II molar relationship and an overjet > 2 mm of the maxillary incisors. Class II.2 was defined by at least an end-to-end class II molar relationship and an overjet ≤ 2 mm of at least two maxillary incisors. Class III was defined by the mesiobuccal cusp of the maxillary first molar occluding at least more than a half cusp distally to the mandibular first molar’s mesiobuccal groove. Lateral crossbite was noted when at least one premolar and/or molar along with its antagonists was affected. Horizontal overlap (overjet) and vertical overlap (overbite) were measured clinically with a stainless steel caliper (Munich model, DENTAURUM GmbH & Co. KG, Ispringen, Germany). The caliper had a total length of 15 cm, with a 0.5 mm graduation on one side and a 1 mm scale on the reverse. Measurements were performed directly in the patient’s mouth. Overjet was defined as the distance between the labial surfaces of the upper and lower incisors, while overbite was defined as the extent of vertical overlap of the upper incisors over the lower incisors. Increased overjet was defined as > 4 mm between the labial surfaces of the upper and lower incisors. Increased overbite was noted when the upper incisors overlapped the lower incisors by > 4 mm.

Furthermore, patients were clinically examined using the Diagnostic Criteria for Temporomandibular Disorders (DC/TMD) and diagnoses were made based on the presence of painful temporomandibular disorders, including TMD with combined muscle and joint pain; TMD with muscle pain, and TMD with joint pain [31].

### Polysomnographic sleep and respiratory recordings

Each patient underwent a single-night polysomnography (PSG) recording (Sonata, Löwenstein Medicals, Bad Ems, Germany) in accordance with AASM standards [2]. The recording variables included surface electrodes at the following locations: electroencephalograms (EEG) at C3-M2, C4-M1, O1-M2 and O2-M1, an electrocardiogram (ECG), bilateral electrooculogram (EOG) and electromyograms (EMG) of the submental and tibialis muscle. Additional, EMG electrodes of bilateral masseter muscle were used. Airflow was monitored with oro-nasal thermistors and nasal prongs, chest and abdominal inductive plethysmography (two channels) was measured and SpO_2_ was measured with pulse oximetry (one channel). Sleep stages and respiratory events were scored visually according to current AASM criteria [35]. The following variables were calculated by Miniscreen SW (Löwenstein Medical, Bad, Ems, Germany): percentage of time spent in sleep stages N1, N2, N3, and REM (time spent in each sleep stage/total sleep time*100%), total sleep time (TST: minutes asleep in bed after “ lights off ”, considering only nighttime sleep), apnea hypopnea index per hour sleep (AHI/h), respiratory disturbance index per hour sleep (RDI/h).

### Electromyographic muscle tone and sleep bruxism episodes recording

Bilateral surface electrodes for EMG were placed on the primary muscle belly of the left and right masseter muscles by a trained sleep technician. The accurate positioning on the cheek was ascertained when the patient applied substantial occlusal force. The EMG electrodes were calibrated using three maximum voluntary contractions (MCVs) before initiating the PSG recording (prior to “lights-off”).

**Fig. 1:**
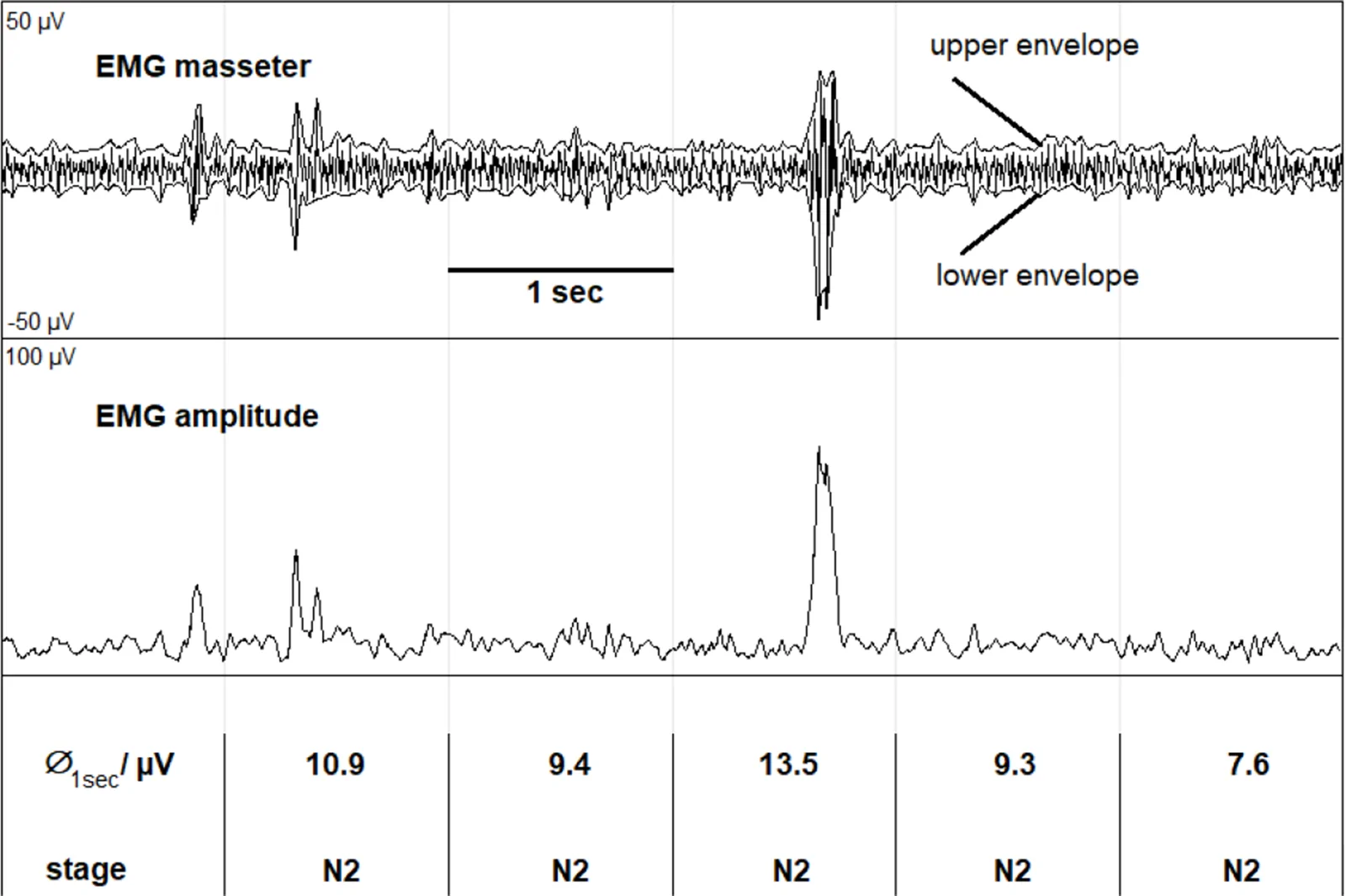
Upper and lower envelopes of the masseter EMG were subtracted to calculate the EMG amplitude. EMG tone values (∅_1sec_) were calculated by averaging the EMG amplitude within each 1-second interval. Mean muscle tone values were calculated subsequently for each sleep stages (N1-N3, REM) [24]

Masseter EMG tone was calculated using automated computer analysis software. The EMG signals were filtered with a band-pass (10–100 Hz) and a 50 Hz notch filter to eliminate mains interference. An adaptive ECG artifact filter was employed when necessary. The automatic analysis calculated the muscle tone through the following steps: First, the upper and lower envelopes of the EMG were calculated and averaged over 5 individual samples (corresponding to 25 ms for an EMG sampled at 200 Hz). The envelopes were subtracted to determine the EMG amplitude shown in Fig. 1. Muscle tone values were calculated as the mean amplitude within each 1-s interval and listed with the corresponding sleep stages. These values represented the data basis for the calculation of the stage-specific statistical parameters (mean, standard deviation, number) of the masseter EMG tone. Periods with phasic EMG activations were not excluded by the automatic analysis because the overall duration of phasic events was normally very short compared to that of tonic EMG [24]. In this second analysis, we aimed to select the best discriminant cut-off point of EMG muscle tone to predict SB.

The EMG electrodes also recorded the number of bruxism episodes per hour (BEI). A BEI was classified if the surface electromyographic burst (bilaterally from the masseter) was greater than 10% of the MVC and if it followed with an interval of 1–5 s and a 20% increase in heart rate with respect to the baseline [36].

### Data analysis

Data analysis was performed using IBM SPSS Statistics (Version 29.0, IBM Corp.). Descriptive statistics were performed for the demographic characteristics of patients. To test for normality of distribution the Kolmogorov-Smirnov test was performed on all variables. Frequencies were expressed as percentage values and frequency rates for occlusion and TMD were compared using Fisher’s exact test.

Mann-Whitney *U* tests were performed for intergroup comparison of jaw functional limitation, for comparison between TMD and jaw functional limitation, as well as between occlusal parameters and jaw functional limitation.

A Receiver Operating Characteristic (ROC) was generated for EMG muscle tone, and the Area Under the Curve (AUC) was calculated to evaluate the quality of this characteristic. To determine an appropriate cut-off point for classifying patients with SB and NSB, the sensitivity (percentage of SB) and specificity (percentage of NSB) of EMG muscle tone values were analyzed [37]. The Spearman correlation was calculated to measure linear correlations between jaw functional limitation and mean EMG muscle tone (mean of measurements of right and left electrode for all EMG tonus measurements in TST). Statistical significance was set at *P* < 0.05.

## Results

39 patients (mean age = 54.4 ± 12.6 years, mean body mass index (BMI) = 31.4 ± 5.8 kg/m^2^, 25 males, 14 females, AHI = 30.3 ± 20.6, RDI = 36.6 ± 18.9) were allocated to the SB group, while 66 patients (mean age = 57.7 ± 10.5 years, mean BMI = 30.9 ± 6.9 kg/m^2^, 49 males, 17 females, AHI = 26.9 ± 21.6, RDI = 32.8 ± 20.9) were allocated to the NSB group. The SB group showed a mean BEI of 9.4/h and the NSB group showed a mean BEI of 2.7/h. The mean time spent in sleep stages, as well as TST, did not differ significantly between the two groups (SB: N1: 6.49 ± 6.72%, N2: 50.78 ± 9.86%, N3: 25.23 ± 10.95%, REM: 17.48 ± 5.88%, TST: 391.75 ± 65.56 min.; NSB: N1: 6.39 ± 4.90%, N2: 51.23 ± 12.19%, N3: 24.88 ± 10.88%, REM: 17.50 ± 7.74%, TST: 378.97 ± 66.16 min.)

### Intergroup comparison of jaw functional limitation

**Fig. 2:**
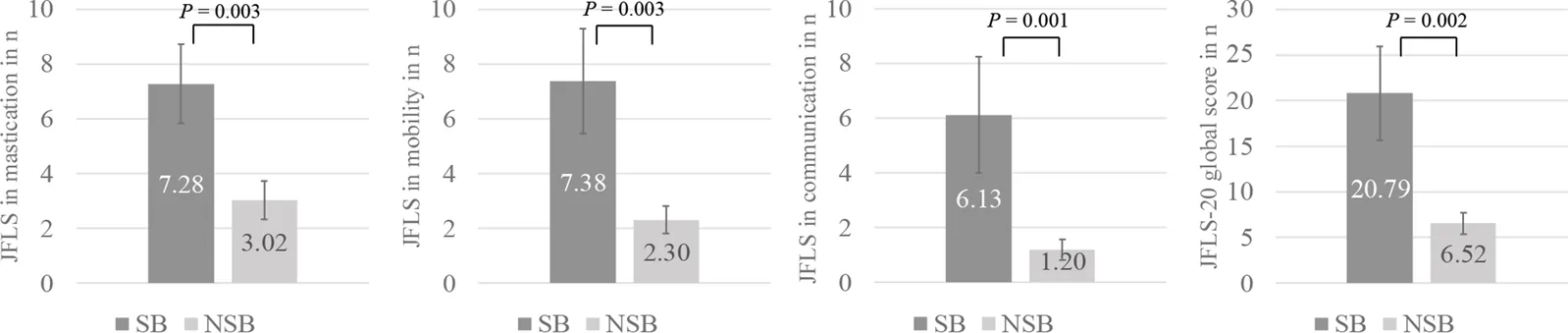
Differences in jaw functional limitation (JFLS) subscales mastication, mobility, communication and the global score (JFLS-20) between sleep bruxism (SB: *n* = 39) and non-sleep bruxism (NSB: *n* = 66) patients. Data are shown as mean ± standard error of the mean (SEM). *P* value was performed using Mann-Whitney *U* tests

The SB group showed significant higher limitations in mastication (*P* = 0.003), mobility (*P* = 0.003) and communication (*P* = 0.001), as well as in the global score of JFLS-20 (*P* = 0.002) compared to the NSB group (Table 2; Fig. 2).

**Table 2:** Differences in jaw functional limitation between sleep bruxism and non-sleep bruxism patients

	SB (37.1%, *n* = 39)	NSB (62.9%, *n* = 66)	
JFLS subscale scores	Mean ± SD		Intergroup comparison *P* value^a^
Limitation in mastication	7.28 ± 8.99	3.02 ± 5.73	0.003**
Limitation in mobility	7.38 ± 11.98	2.30 ± 4.07	0.003**
Limitation in communication	6.13 ± 13.25	1.20 ± 3.01	0.001**
**Global Score**	20.79 ± 31.96	6.52 ± 9.70	0.002**

^a^ *P* value was performed using Mann-Whitney *U* tests (** *P* < 0.01, * *P* < 0.05). SD = standard deviation

Abbreviations: SB = Sleep-bruxism group, NSB = No sleep-bruxism group, JFLS-20 = Jaw Functional Limitation Scale with 20 items, SD = standard deviation

### Intergroup comparison of occlusal results

**Fig. 3:**
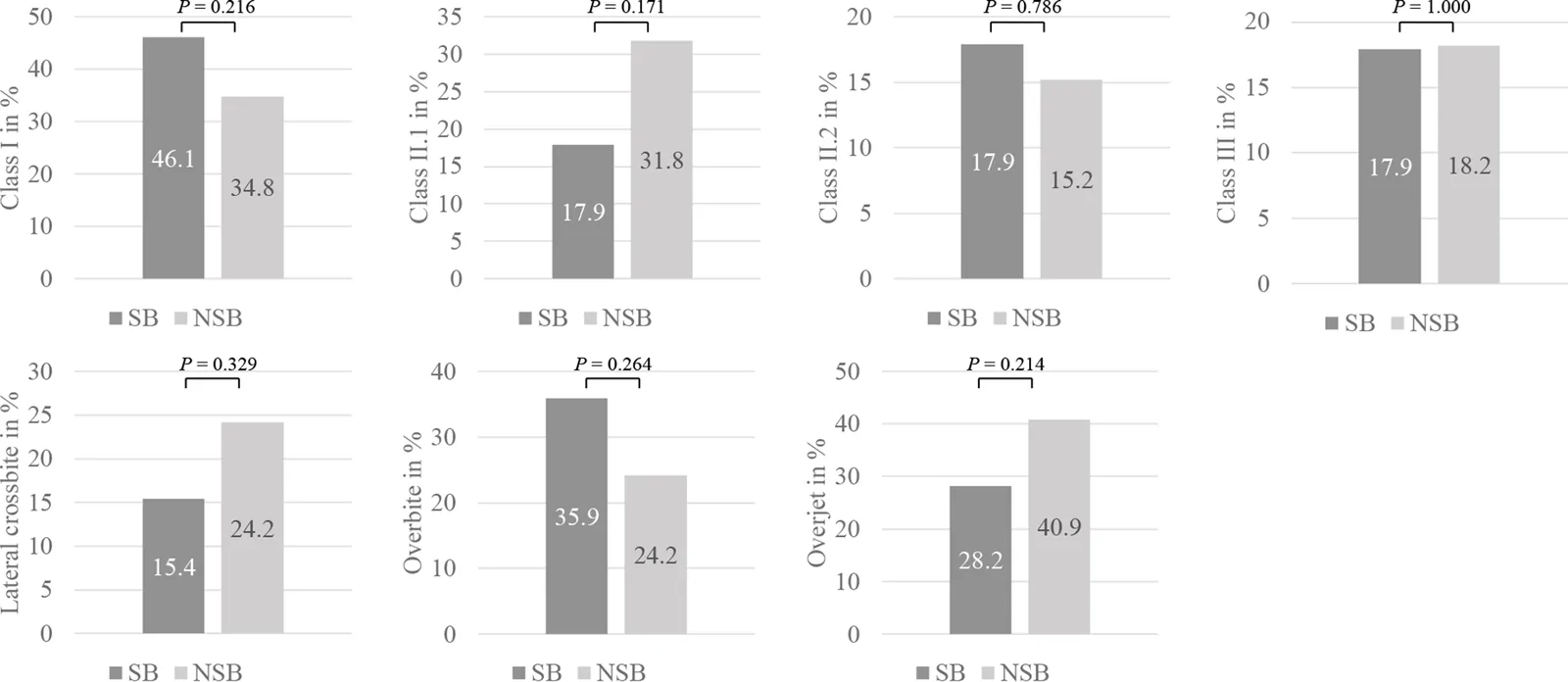
Frequencies of dento-skeletal class I, class II.1, class II.2, class III between sleep bruxers (SB: *n* = 39) and no sleep bruxers (NSB: *n* = 66) and of the occlusal parameters lateral crossbite, increased overbite, increased overjet between both groups (SB: *n* = 39, NSB: *n* = 66). *P* value was performed using Fisher’s exact test

The frequencies of the occlusal parameters (class I, class II.1, class II.2, class III; lateral crossbite, increased overjet and overbite) are shown in Fig. 3. Intergroup comparison revealed no significant differences in any of the occlusal parameters.

### Intergroup comparison of TMD symptom results

The prevalence of painful muscle and joint TMD between the SB and NSB group is shown in Table 3. The SB group exhibited a significantly higher prevalence of painful muscle TMD (*P* = 0.01) compared to the NSB group.

**Table 3:** Prevalence of the painful muscle and joint temporomandibular disorders between patients with sleep bruxism and without sleep bruxism

	Patients with SB (37.1%, *n* = 39)	Patients without SB (62.9%, *n* = 66)		
Diagnosis of TMD			*P* value^a^	
TMD with muscle and joint pain	61.5% (23)	21.2% (13)	< 0.001***	
TMD with muscle pain	46.1% (16)	16.7% (11)	0.01*	
TMD with joint pain	15.4% (7)	4.5% (2)	0.074	

Abbreviations: SB = sleep bruxism, TMD = Temporomandibular disorder

^a^ *P* value was performed using Fisher’s exact test (*** *P* < 0.001, * *P* < 0.05)

### EMG muscle tone

The mean EMG muscle tone was lowest during REM sleep (SB: 10.17 ± 0.65 µV; NSB: 8.98 ± 1.00 µV) and highest during sleep stage N1 (SB: 11.04 ± 0.78 µV; NSB: 9.33 ± 1.21 µV). In sleep stage N2, the mean values were 10.81 ± 0.94 µV for the SB group and 9.16 ± 1.10 µV for the NSB group. Sleep stage N3 showed mean values of 10.57 ± 1.17 µV in the SB group and 9.10 ± 1.10 µV in the NSB group. Across all sleep stages, the mean EMG muscle tone was significantly higher in the SB group (*P* < 0.001).

**Fig. 4:**
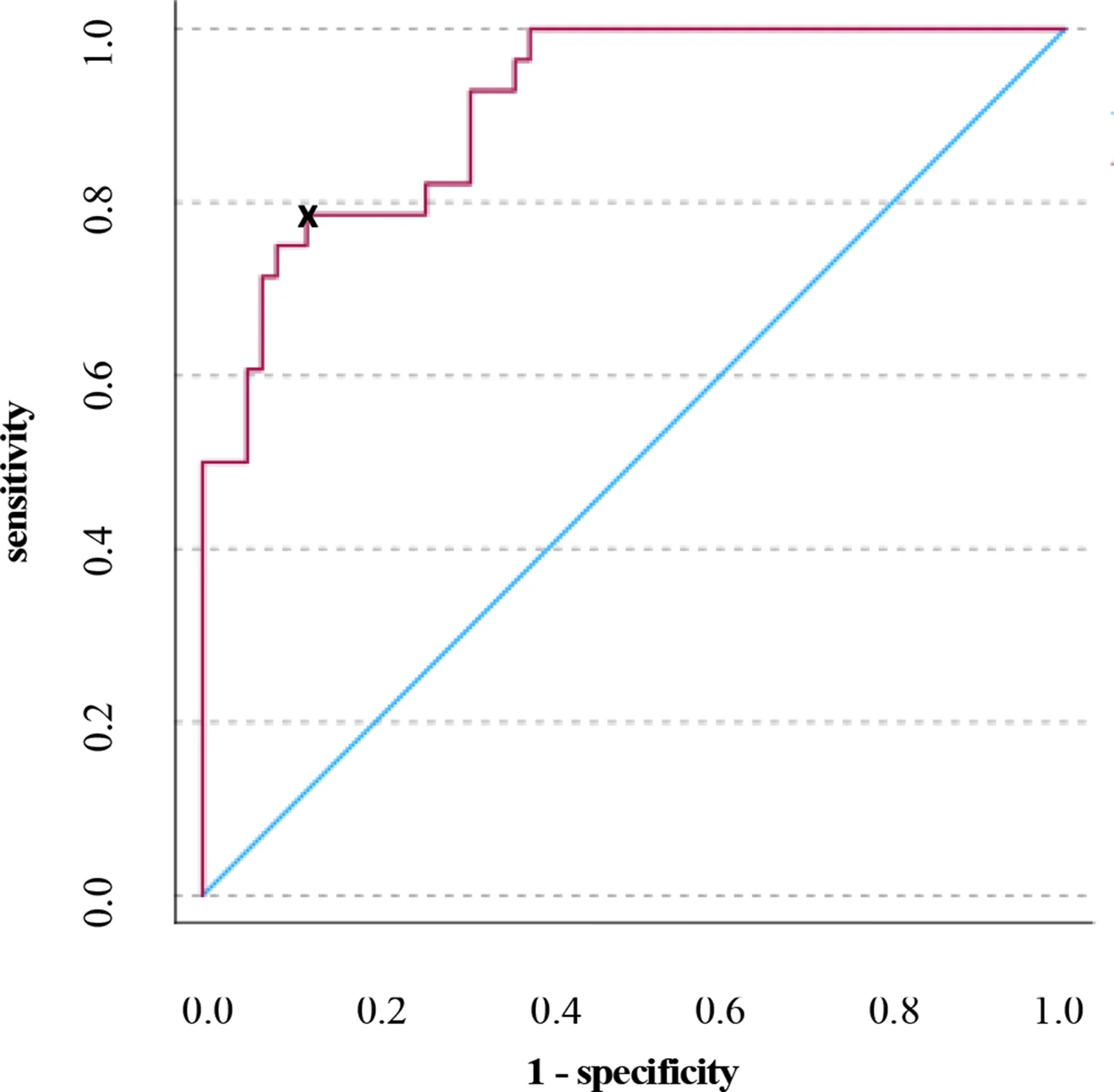
Receiver Operating Characteristic (ROC) to select the best discriminant cut-off point of EMG muscle tone to classify between sleep bruxers and non-sleep bruxers

Figure 4 presented the ROC curve. The AUC was 0.911 (*P* < 0.001). An EMG muscle tone value of 9.79 µV showed a sensitivity of 78.6% and a specificity of 87.9% and was selected as the best discriminant cut-off point.

### Functional and occlusal results in total OSA patients

#### Jaw functional limitation, TMD and occlusion

Considering jaw functional limitation and TMD, there was a significant difference between TMD and NTMD in the subscales mastication (*P* < 0.003), mobility (*P* < 0.001), communication (*P* = 0.011) and the global score (*P* < 0.001). However, when considering jaw functional limitation and occlusion, no significant differences were observed between them (Table 4).

**Table 4:** Differences between jaw functional limitation and the occlusal parameters (class I, class II.1, class II.2, class III, cross bite, overjet, overbite)

	**Patients with TMD** (36.2%, *n* = 38)	**Patients without TMD** (63.8%, *n* = 67)	
**JFLS-20 subscales**			***P*** **– value**
Limitation in mastication	7.58 ± 9.15	2.91 ± 5.54	0.003**
Limitation in mobility	8.66 ± 12.11	1.66 ± 2.88	< 0.001***
Limitation in communication	6.47 ± 13.52	1.07 ± 2.43	0.011*
**JFLS-20 global score**	22.71 ± 31.93	5.64 ± 8.83	< 0.001***
	**Patients with cross bite** (21%, *n* = 22)	**Patients without cross bite** (79%, *n* = 83)	
**JFLS-20 subscales**			***P*** **– value**
Limitation in mastication	4.45 ± 6.95	4.64 ± 7.52	0.635
Limitation in mobility	4.27 ± 6.48	4.17 ± 8.75	0.810
Limitation in communication	2.14 ± 3.27	3.27 ± 9.64	0.174
**JFLS-20 global score**	10.86 ± 13.50	12.07 ± 23.70	0.375
	**Patients with Class I** (40%, *n* = 42)	**Patients without Class I** (60%, *n* = 63)	
**JFLS-20 subscales**			***P*** **– value**
Limitation in mastication	5.26 ± 8.26	4.16 ± 6.73	0.795
Limitation in mobility	5.55 ± 11.33	3.29 ± 5.36	0.913
Limitation in communication	4.55 ± 12.21	2.02 ± 5.06	0.601
**JFLS-20 global score**	15.36 ± 30.08	9.46 ± 13.90	0.963
	**Patients with Class II.1** (26.7%, *n* = 28)	**Patients without Class II.1** (73.3%, *n* = 77)	
**JFLS-20 subscales**			***P*** **– value**
Limitation in mastication	4.32 ± 7.54	4.70 ± 7.36	0.682
Limitation in mobility	2.79 ± 3.65	4.70 ± 9.41	0.762
Limitation in communication	1.50 ± 3.72	3.58 ± 9.87	0.434
**JFLS-20 global score**	8.61 ± 11.04	12.99 ± 24.67	0.935
	**Patients with Class II.2** (16.2%, *n* = 17)	**Patients without Class II.2** (83.8%, *n* = 88)	
**JFLS-20 subscales**			***P*** **– value**
Limitation in mastication	4.06 ± 5.82	4.70 ± 7.66	0.667
Limitation in mobility	5.12 ± 7.92	4.01 ± 8.40	0.391
Limitation in communication	3.24 ± 8.26	2.99 ± 8.82	0.722
**JFLS-20 global score**	12.41 ± 19.91	11.70 ± 22.38	0.569
	**Patients with Class III** (18.1%, *n* = 19)	**Patients without Class III** (81.9%, *n* = 86)	
**JFLS-20 subscales**			***P*** **– value**
Limitation in mastication	3.79 ± 6.43	4.78 ± 7.58	0.622
Limitation in mobility	2.53 ± 4.40	4.56 ± 8.91	0.456
Limitation in communication	1.58 ± 2.24	3.35 ± 9.53	0.680
**JFLS-20 global score**	7.89 ± 10.87	12.70 ± 23.62	0.730
	**Patients with overbite ≥ 4 mm** (28.6%, *n* = 30)	**Patients with overbite < 4 mm** (71.4%, *n* = 75)	
**JFLS-20 subscales**			***P*** **– value**
Limitation in mastication	5.03 ± 8.88	4.43 ± 6.73	0.963
Limitation in mobility	5.03 ± 9.83	3.85 ± 7.65	0.645
Limitation in communication	4.00 ± 9.60	2.64 ± 8.34	0.144
**JFLS-20 global score**	14.07 ± 27.06	10.92 ± 19.62	0.902
	**Patients with overjet ≥ 4 mm** (36.2%, *n* = 38)	**Patients with overjet < 4 mm** (63.8%, *n* = 67)	
**JFLS-20 subscales**			***P*** **– value**
Limitation in mastication	5.26 ± 9.05	4.22 ± 6.30	0.965
Limitation in mobility	4.29 ± 8.70	4.13 ± 8.15	0.780
Limitation in communication	2.82 ± 8.70	3.15 ± 8.80	0.563
**JFLS-20 global score**	12.37 ± 23.87	11.51 ± 20.90	0.910

Abbreviations: TMD = painful temporomandibular Disorder; JFLS-20 = Jaw Functional Limitation Scale with 20 items

JFLS-20 subscales and global score were measured using means and standard deviation

*P* value was analyzed using Mann-Whitney *U* tests (*** *P* < 0.001, ** *P* < 0.01, * *P* < 0.05)

### Correlation between jaw functional limitation and muscle tone

**Fig. 5:**
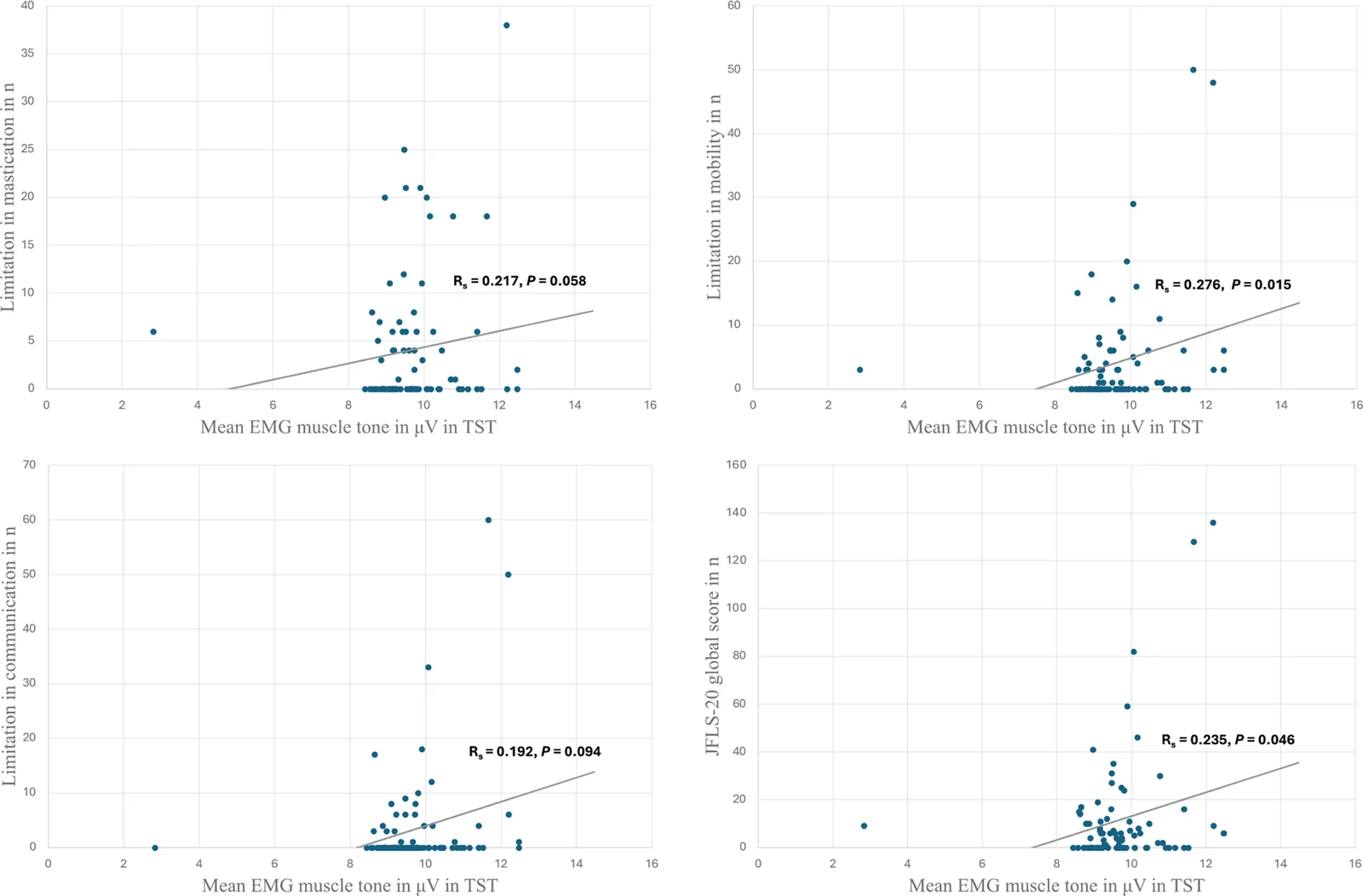
Spearman correlation coefficient (rs) was calculated to measure linear correlations between jaw functional limitation (JFLS) subscales mastication, mobility, communication and the global score (JFLS-20) and mean EMG (electromyographic) muscle tone in µV in TST (total sleep time [minutes asleep in bed after “lights off”, considering only nighttime sleep]). The correlation is two-sided significant at the level of 0.05 (* *P* < 0.05)

The correlation between jaw functional limitation and EMG muscle tone is presented in Table 5; Fig. 5. EMG muscle tone correlated significantly with the subscale mobility (*P* = 0.015) and the global score (*P* = 0.046). However, the correlation with the subscale mastication (*P* = 0.058) and communication (*P* = 0.094) was not significant.

**Table 5:** Correlation between EMG muscle tone and jaw functional limitation (JFLS-20)

	Mean EMG muscle tone (µV; TST)	
**JFLS-20 subscales**	Spearman correlation^a^	*P* value
Limitation in mastication	0.217	0.058
Limitation in mobility	0.276	0.015*
Limitation in communication	0.192	0.094
**JFLS- 20 global score**	0.235	0.046*

^a^ Spearman correlation; the correlation is two-sided significant at the level of 0.05 (* *P* < 0.05)

JFLS-20 = Jaw Functional Limitation Scale (20 items); mean EMG muscle tone = mean electromyographic muscle tone; TST = total sleep time (minutes asleep in bed after “lights off”, considering only nighttime sleep)

## Discussion

This prospective clinical trial aimed to assess the functional and occlusal parameters associated with probable SB in patients with OSA. We hypothesized that functional characteristics in OSA could serve as diagnostic tools to differentiate between SB and NSB rather than relying on static occlusal parameters. The results of this analysis support our hypothesis and are discussed as follows:

The EMG muscle tone analysis in SB patients is a new methodology, we previously published [24]. This analysis is a part of a larger project in sleep apnea patients with and without SB. Additionally, to the best of our knowledge, we were the first to calculate a discriminant cut-off point for EMG muscle tone to predict SB in OSA patients. In our initial analysis we represented the first attempt to measure the average EMG muscle tone in a substantial sample of OSA patients, both with and without probable SB. We demonstrated that EMG muscle tone was consistently higher across all sleep stages in SB compared to those without. In the here presented data a Receiver Operating Characteristic (ROC) analysis was performed. The analysis yielded a favourable Area Under Curve (AUC) result, demonstrating, that EMG muscle tone is a significant predictor of SB, with a value of 9.79 µV identified as the optimal discriminant cut-off point, typically based on the upper left point of the curve [38]. Using this cut-off point, it is predicted that 78.6% of the patients with SB will be correctly classified as having SB, while 87.9% of patients without SB will be accurately identified as NSB patients. However, it should be in mind, that this cut-off point is not an absolute value and should not be utilized without a comprehensive patient clinical examination [38, 39]. Regarding the shape of the curve, using a cut-off value of 9.66 µV would increase the sensitivity to 82% but decrease the specificity to 84.1%. This implies that more patients without SB would be incorrectly classified as having SB. Conversely, using a cut-off value of 9.88 µV would reduce the sensitivity to 75% and increase specificity to 91.4%.

Additionally, we were the first to analyze the JFLS-20 subscale scores and their correlation with EMG muscle tone in this large cohort of OSA patients, both with and without SB. Our findings revealed that self-reported limitations in mastication, mobility, and communication were significantly higher in SB patients compared to those without SB. Chantaracherd et al. [40] reported a mean JFLS-20 global score of about 30 for patients with TMD. In contrast, our results showed slightly lower scores of about 20 for SB patients and 22 for patients with painful TMD. One possible explanation for the nearly similar scores is that SB may also lead to orofacial muscle symptoms, such as stiffness or soreness of the orofacial muscles rather than pain. Additionally, the prevalence of painful TMD symptoms was high in the SB group (62.5%), which may contribute to similar self-reported jaw functional limitations observed. Limitations in mobility and overall jaw function were positively correlated with EMG muscle tone (Fig. 5). The correlation between limited jaw mobility and EMG muscle tone may be explained by the fact that SB patients often exhibit masseter muscle hypertrophy, a condition frequently associated with symptoms such as limited mouth opening and increased muscle tension in this area.

Although there is a growing consensus that occlusion is not a causative factor for SB [13, 41] our results indicate that occlusal parameters did not significantly differ between OSA patients with and without SB. Furthermore, there were no notable differences in limitations in mastication, mobility and communication. Most studies that evaluated occlusion as an etiological factor rely on patient history or questionnaires. The results obtained are often inconsistent [42, 43].

While some authors reported that occlusal factors do not play a significant role in triggering SB, others identified an association between SB and malocclusion, including studies that relied on questionnaires without clinical examination [42, 44–46]. Occlusal factors, such as deep bite or posterior forced bite, commonly seen in Class II.2, or lateral forced bite, have been traditionally considered causal factors for SB. It was believed that patients attempt to eliminate these occlusal interferences through grinding movements of the mandible. Lavigne et al. [13] highlighted that tooth contact occurs only for approximately 17.5 min in 24 h, suggesting that clinicians often overestimate the role of occlusal factors. Sleep laboratory studies have shown that SB-related muscle activity lasted approximately 8 min over a typical sleep period of 7 to 9 h. Importantly, during these 8 min of muscle activity, tooth contact is not always present, often, it is merely a tension of the masseter muscle. This understanding, combined with the observation that corrective occlusal adjustments do not eliminate SB, has led to a shift away from viewing occlusion as a primary causative factor. The notion that occlusal factors do not promote SB is further supported by the evidence that the treatment of OSA with continuous positive airway pressure (CPAP) masks or mandibular advancement devices (MADs) reduces the frequency of SB events, suggesting an etiological functional links between SB and OSA [47, 48].

Given that inadequate activity of upper airway muscles during sleep, is a major contributor to OSA, it has been hypothesized that nocturnal muscle activation may serves as a compensatory mechanism to maintain upper airway patency [24, 49]. Our findings of increased nocturnal muscle tone in the SB group may support this hypothesis. Nevertheless, this hypothesis remains unproven and further clinical studies are needed to validate these findings.

### Limitations of the study

Despite the strengths of our study, which calculated a cut-off value for EMG muscle tone, several limitations must be acknowledged. First, due to its cross-sectional design, causal relationships between SB and the functional or occlusal findings described cannot be established. Second, the diagnosis of SB was based on clinical criteria for “probable” SB rather than on PSG-confirmed diagnosis, which may have influenced the results.

Furthermore, we did not assess the BEI in greater detail, although our SB group exhibited a significantly elevated mean BEI. Follow-up assessments conducted within the framework of a separate project revealed considerable variability in both the BEI and rhythmic masticatory muscle activity (RMMA) events as previously described by Ohlmann et al. [20] and Lavigne et al. [19]. This variability led us to shift our focus to the analysis of mean EMG muscle tone. Since the EMG muscle tone analysis represents a new methodology, it remains unclear to what extent such variability may have affected our results. It is therefore possible that similar night-to-night fluctuations may also occur in EMG muscle tone, and future studies incorporating multi-night PSG recordings are necessary to better understand this potential variability and its implications for clinical assessment.

The cut-off value of 9.79 µV, although supported by our ROC analysis, may have limited validity due to potential confounding factors that were not fully controlled within our sample, such as age, sex, and TMD. These variables can independently affect muscle tone and may reduce the specificity and sensitivity of the proposed threshold. For example, patients with TMD may exhibit elevated muscle tone without the presence of SB, leading to possible false positives. Thus, the cut-off value may serve as a preliminary reference point rather than a definitive criterion and must always be interpreted in the context of a comprehensive clinical examination, pending further external validation.

Another limitation concerns the classification of skeletal classes I, II, and III. A reliable evaluation of skeletal classification requires cephalometric analysis based on lateral cephalometric radiographs. However, within the context of our study, the indication for lateral radiographs did not meet ethical justification, and the institutional ethics committee did not grant approval, adhering to the ALARA principle (“As Low As Reasonably Achievable”). To address potential discrepancies between dental and skeletal classifications - particularly in cases with suspected mesial migration of the first molars - we applied the reconstruction method described by Grünberg [34] in selected cases. This orthodontic approach permits an estimation of the original position of the first molars when current alignment has been altered due to tooth loss or crowding.

From an orthodontic perspective, this method approximates the original occlusal relationship and generally provides a reasonable estimate of the corresponding skeletal class (I, II, or III). It represents an established and routinely applied procedure in orthodontics, commonly used for clinical diagnostics and treatment planning. Nevertheless, we would like to emphasize that this classification represents a dental approximation and does not constitute a cephalometrically validated skeletal analysis. Although occlusal class was used as a proxy for skeletal classification, we acknowledge that this approach cannot replace cephalometric radiographs in accurately assessing craniofacial morphology. The rationale for applying this method in our study was to investigate whether patients with Class II malocclusion - particularly those with Class II Division 2 - attempt to compensate for a presumed retral forced bite by posturing the mandible forward, potentially resulting in a wider pharyngeal airway. We recognize that this method does not allow for an exact assessment of the skeletal anteroposterior relationship and does not provide precise angular measurements such as the ANB angle or Wits appraisal.

As a general limitation of our patient sample is that older patients tend to have poorer dental conditions compared to younger patients. Oghli et al. [16] found that patients with prosthetic rehabilitation had significantly higher JFLS values. Poorer dental conditions may predispose individuals to impaired jaw function. In our cohort, patients with SB were slightly younger on average, though the age difference was not significant. Therefore, we assume that the dental condition between the two groups was comparable. Despite the limitations inherent in a questionnaire-based approach, the JFLS-20 questionnaire may be useful additional diagnostic tool for identifying SB patients, who exhibited significantly higher subscale and total scores compared to patients without SB [32, 33, 50].

Despite its limitations, the present study demonstrates several noteworthy strengths. This prospective clinical study introduces a novel quantitative approach to evaluating EMG muscle tone in the context of probable SB among OSA patients. The identification of a predictive cut-off value has been shown to offer an additional clinically relevant tool to aid in the differentiation of SB from NSB. Further investigation of the EMG muscle tone is required in future clinical studies, in order to validate this cut-off point.

## Conclusion

The results indicate that occlusal parameters do not possess diagnostic value. However, an EMG muscle tone could be an additional, clinically reliable tool, as we have shown that 9.79 µV can serve as a preliminary cutoff point for distinguishing SB from NSB in patients with OSA, pending external validation.

Additional functional factors, including self-reported jaw functional limitations in mastication, mobility, and verbal and expressive communication, as well as a higher prevalence of painful muscle TMD symptoms, may further support the identification of SB in this population. Occlusal characteristics, however, do not appear to differ significantly between groups, nor do they seem to contribute substantially to jaw functional limitations within the overall OSA cohort. Taken together, and in line with existing evidence, these results tentatively support the hypothesis that SB in OSA patients could represent a compensatory muscular adaptation, potentially responsive to sleep apnea therapy. Further studies are warranted to confirm these findings and to establish the clinical utility of EMG muscle tone measurements in this context.

## Data Availability

No datasets were generated or analysed during the current study.
